# 1-Benzhydryl-4-(4-chloro­phenyl­sulfonyl)piperazine

**DOI:** 10.1107/S1600536807067888

**Published:** 2008-01-04

**Authors:** H. R. Girisha, S. Naveen, K. Vinaya, M. A. Sridhar, J. Shashidhara Prasad, K. S. Rangappa

**Affiliations:** a Department of Studies in Chemistry, Mansagangotri, University of Mysore, Mysore 570 006, India; b Department of Studies in Physics, Mansagangotri, University of Mysore, Mysore 570 006, India

## Abstract

The title compound, C_23_H_23_ClN_2_O_2_S, was synthesized by the nucleophilic substitution of 1-benzhydrylpiperazine with 4-chloro­phenyl­sulfonyl chloride. The piperazine ring is in a chair conformation. The geometry around the S atom is that of a distorted tetra­hedron. There is a large range of bond angles around the piperazine N atoms. The dihedral angle between the least-squares plane (p1) defined by the four coplanar C atoms of the piperazine ring and the benzene ring is 81.6 (1)°. The dihedral angles between p1 and the phenyl rings are 76.2 (1) and 72.9 (2)°. The two phenyl rings make a dihedral angle of 65.9 (1)°. Intramolecular C—H⋯O hydrogen bonds are present.

## Related literature

For related literature, see: Bassindale (1984 ▶); Berkheij *et al.* (2005 ▶); Campbell *et al.* (1973 ▶); Cremer & Pople (1975 ▶); Dinsmore & Beshore (2002 ▶); Humle & Cherrier (1999 ▶); Katzung (1995 ▶).
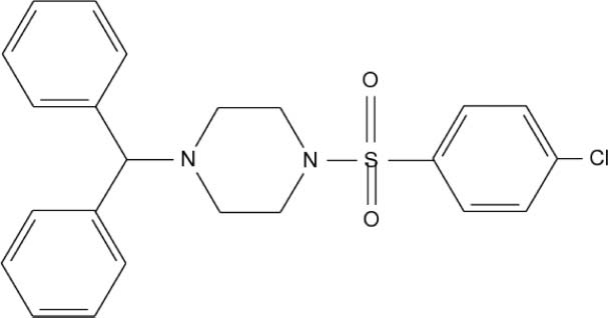


## Experimental

### 

#### Crystal data

C_23_H_23_ClN_2_O_2_S
*M* *_r_* = 426.94
Monoclinic, 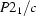

*a* = 9.392 (7) Å
*b* = 13.114 (10) Å
*c* = 19.225 (11) Å
β = 113.645 (3)°
*V* = 2169 (3) Å^3^
*Z* = 4
Mo *K*α radiation
μ = 0.29 mm^−1^
*T* = 295 (2) K
0.25 × 0.20 × 0.20 mm

#### Data collection

MacScience DIPLabo 32001 diffractometer
Absorption correction: none
7255 measured reflections
3818 independent reflections
2917 reflections with *I* > 2σ(*I*)
*R*_int_ = 0.024

#### Refinement

*R*[*F* ^2^ > 2σ(*F* ^2^)] = 0.051
*wR*(*F* ^2^) = 0.147
*S* = 1.08
3818 reflections
263 parameters
H-atom parameters constrained
Δρ_max_ = 0.33 e Å^−3^
Δρ_min_ = −0.27 e Å^−3^

### 

Data collection: *XPRESS* (MacScience, 2002 ▶); cell refinement: *SCALEPACK* (Otwinowski & Minor, 1997 ▶); data reduction: *DENZO* and *SCALEPACK* (Otwinowski & Minor, 1997 ▶); program(s) used to solve structure: *SHELXS97* (Sheldrick, 1997 ▶); program(s) used to refine structure: *SHELXL97* (Sheldrick, 1997 ▶); molecular graphics: *PLATON* (Spek, 2003 ▶) and *ORTEPII* (Johnson, 1976 ▶); software used to prepare material for publication: *PLATON*.

## Supplementary Material

Crystal structure: contains datablocks I, global. DOI: 10.1107/S1600536807067888/hg2365sup1.cif

Structure factors: contains datablocks I. DOI: 10.1107/S1600536807067888/hg2365Isup2.hkl

Additional supplementary materials:  crystallographic information; 3D view; checkCIF report

## Figures and Tables

**Table 1:** Hydrogen-bond geometry (Å, °)

*D*—H⋯*A*	*D*—H	H⋯*A*	*D*⋯*A*	*D*—H⋯*A*
C2—H2*A*⋯O8	0.97	2.49	2.890 (3)	105
C6—H6*B*⋯O9	0.97	2.56	2.965 (3)	105
C11—H11⋯O9	0.93	2.53	2.905 (3)	104

**Fig. 1.**
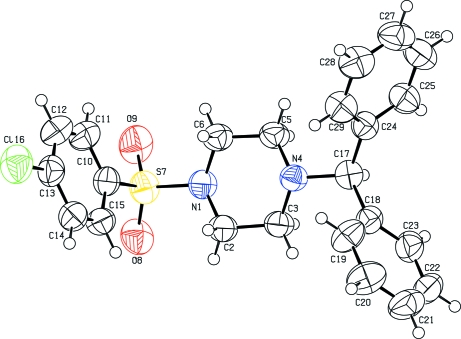
The molecular structure, with atomic numbering scheme. Displacement ellipsoids are drawn at the 50% probability level. H atoms are represented as small spheres of arbitrary radius.

## supplementary crystallographic information

### Comment

Piperazines are among the most important building blocks in today's drug discovery. The piperazine nucleus is capable of binding to multiple receptors with high affinity and therefore piperazine has been classified as a privileged structure (Dinsmore *et al.*, 2002). They are found in biologically active compounds across a number of different therapeutic areas (Berkheij *et al.*, 2005) such as antifungal, antibacterial, antimalarial, antipsychotic, antidepressant and antitumour activity against colon, prostate, breast, lung and leukemia tumors (Humle & Cherrier, 1999). 1-Benzylpiperazine was originally synthesized as a potential antihelminthic (Campbell *et al.*, 1973) and these derivatives were found to possess excellent pharmacological activities such as vasodilator, hypotensive, antiviral activity and cerebral blood flow increasing actions, broad pharmacological action on central nerves system (*CNS*), especially on dopaminergic neurotransmission. Sulfonamides are among the most widely used antibacterial agents (Katzung *et al.*, 1995). Piperazine sulfonamides exhibit diverse therapeutic activity such as antibacterial activity, MMP-3 inhibition and carbonic anhydrase inhibition. Encouraged by the above information, the title compound was synthesized and herein we report its crystal structure.

A perspective view of the title compound is shown in Fig. 1. A study of torsion angles, asymmetry parameters and least-squares plane calculations reveal that the piperazine ring in the structure is in a chair conformation. This has been confirmed by the puckering paramaters q_2_=0.0291 (24) Å, q_3_=0.5969 (26) Å, Q_T_=0.5977 (26) Å, θ=3.07 (23)° and φ=198 (5)° (Cremer & Pople, 1975). The conformation of the attachment of the diphenylmethyl and the sulfonyl groups to the piperazine ring are best described by the torsion angle values of 166.6 (2)° and -177.4 (2)° for S7—N1—C2—C3 and C17—N4—C5—C6, respectively; *i.e.* they adopt +antiperiplanar and -antiperiplanar conformations, respectively. The bonds N1—S7 and N4—C17 connecting the sulfonyl and the diphenyl groups make angles of 86.00 (11)° and 72.92 (14)°, respectively, with the Cremer and Pople plane of the piperazine ring and thus are in the equatorial plane of the piperazine ring.

The bond angles about the S atom shows significant deviation from that of a regular tetrahedron, with the largest deviations being observed for O9—S7—O8 [119.92 (12)°] and 09—S7—C10 [107.88 (12)°]. The widening of O8—S7—O9 is due to the repulsive interactions between the S?O bonds and the non-bonded interactions involving the two S?O bonds and the varied steric hindrance of the substituents. The structure thus has less steric interference. The reduction of the N1—S7—C10 angle from the ideal tetrahedral value is attributed to the Thorpe-Ingold effect (Bassindale, 1984). The sulfonyl O atoms, O8 and O9, are oriented in -synclinal and +synclinal conformations, respectively, as indicated by the torsion angle values of -42.1 (2)° and 53.96 (19)° for C2—N1—S7—O8 and C6—N1—S7—O9, respectively.

### Experimental

A solution of 1-benzhydryl-piperazine (0.5 g, 1.98 mmol) in dry dichloromethane was taken, and cooled to 0–5° C in an ice bath. Then triethylamine (0.601 g, 5.94 mmol) was added to the cold reaction mixture and stirred for 10 minutes. Then 4-chloro-benzenesulfonyl chloride (0.417 g, 1.98 mmol) was added. The reaction mixture was stirred at room temperature for 5 hrs. The reaction mixture was monitored by TLC. On completion of the reaction, the solvent was removed under reduced pressure and the residue was taken in water and extracted with ethyl acetate. Finally water wash was given to organic layer and dried with anhydrous sodium sulfate. The solvent was evaporated to get crude product, which was purified by column chromatography over silica gel using hexane: ethyl acetate (8:2) as an eluent. Pure white crystals were obtained due to the slow evaporation of the solvent with a yield of 90%. *M*.p. 428.1 K.

^1^HNMR (DMSO, 400 MHz): δ 7.7–7.8 (m, 4H, Ar—H), 7.4 (d, 4H, Ar—H), 7.25(t, 4H, Ar—H), 7.16 (t, 2H, Ar—H), 4.32 (s, 1H, –CH), 3.32 (dd, 4H, –CH_2_), 2.41 (dd, 4H, –CH_2_).

MS (ESI + ion): m/*z* = 427.9

IR (KBr, cm^1^): 2961, 2889, 1350, 1279, 707.

Anal. Calcd.for C_23_H_23_ClN_2_O_2_S (in %): C-59.87, H-4.81, N-6.07, S-6.95. Found C-59.82, H-4.78, N-6.04, S-6.90%.

### Refinement

H atoms were placed at idealized positions and allowed to ride on their parent atoms with C—H distances in the range 0.92–0.97 Å and O—H = 0.82 Å; *U*_iso_(H) values were set equal to 1.2*U*_eq_(carrier atom).

### Crystal data

**Table:**

C_23_H_23_ClN_2_O_2_S	*F*_000_ = 896
*M**_r_* = 426.94	*D*_x_ = 1.307 Mg m^−^^3^
Monoclinic, *P*2_1_/*c*	Mo *K*α radiation λ = 0.71073 Å
Hall symbol: -P 2 ybc	Cell parameters from 7255 reflections
*a* = 9.392 (7) Å	θ = 2.3–25.0º
*b* = 13.114 (10) Å	µ = 0.29 mm^−^^1^
*c* = 19.225 (11) Å	*T* = 295 (2) K
β = 113.645 (3)º	Block, white
*V* = 2169 (3) Å^3^	0.25 × 0.20 × 0.20 mm
*Z* = 4	

### Data collection

**Table:**

MacScience DIPLabo 32001 diffractometer	3818 independent reflections
Radiation source: fine-focus sealed tube	2917 reflections with *I* > 2σ(*I*)
Monochromator: graphite	*R*_int_ = 0.024
Detector resolution: 10.0 pixels mm^-1^	θ_max_ = 25.0º
*T* = 295(2) K	θ_min_ = 2.3º
ω scans	*h* = −11→11
Absorption correction: none	*k* = −15→15
7255 measured reflections	*l* = −22→22

### Refinement

**Table:**

Refinement on *F*^2^	Hydrogen site location: inferred from neighbouring sites
Least-squares matrix: full	H-atom parameters constrained
*R*[*F*^2^ > 2σ(*F*^2^)] = 0.051	*w* = 1/[σ^2^(*F*_o_^2^) + (0.0781*P*)^2^ + 0.4503*P*] where *P* = (*F*_o_^2^ + 2*F*_c_^2^)/3
*wR*(*F*^2^) = 0.147	(Δ/σ)_max_ < 0.001
*S* = 1.08	Δρ_max_ = 0.33 e Å^−^^3^
3818 reflections	Δρ_min_ = −0.27 e Å^−^^3^
263 parameters	Extinction correction: SHELXL97 (Sheldrick, 1997), FC^*^=KFC[1+0.001XFC^2^Λ^3^/SIN(2Θ)]^-1/4^
Primary atom site location: structure-invariant direct methods	Extinction coefficient: 0.065 (4)
Secondary atom site location: difference Fourier map	

### Special details

**Table:**

Geometry. Bond distances, angles *etc*. have been calculated using the rounded fractional coordinates. All e.s.d.'s are estimated from the variances of the (full) variance-covariance matrix. The cell e.s.d.'s are taken into account in the estimation of distances, angles and torsion angles
Refinement. Refinement on *F*^2 for ALL reflections except those flagged by the user for potential systematic errors. Weighted *R*-factors *wR* and all goodnesses of fit *S* are based on *F*^2, conventional *R*-factors *R* are based on *F*, with *F* set to zero for negative *F*^2. The observed criterion of *F*^2 > σ(*F*^2) is used only for calculating -*R*-factor-obs *etc*. and is not relevant to the choice of reflections for refinement. *R*-factors based on *F*^2 are statistically about twice as large as those based on *F*, and *R*-factors based on ALL data will be even larger.

### Fractional atomic coordinates and isotropic or equivalent isotropic displacement parameters (Å^2^)

**Table:**

	*x*	*y*	*z*	*U*_iso_*/*U*_eq_	
Cl16	0.45997 (9)	0.44356 (6)	0.31602 (5)	0.0932 (3)	
S7	0.02536 (7)	0.06459 (5)	0.20336 (3)	0.0673 (2)	
O8	−0.12416 (19)	0.08971 (15)	0.20131 (11)	0.0815 (7)	
O9	0.0391 (2)	0.02536 (16)	0.13714 (9)	0.0878 (7)	
N1	0.1012 (2)	−0.02030 (15)	0.27063 (10)	0.0592 (6)	
N4	0.28463 (19)	−0.12983 (14)	0.40601 (10)	0.0540 (6)	
C2	0.0814 (3)	−0.00363 (19)	0.34202 (13)	0.0639 (8)	
C3	0.1236 (3)	−0.09986 (19)	0.38885 (13)	0.0602 (8)	
C5	0.2974 (3)	−0.14861 (19)	0.33344 (12)	0.0611 (8)	
C6	0.2608 (3)	−0.05271 (19)	0.28593 (13)	0.0631 (8)	
C10	0.1441 (3)	0.17362 (19)	0.23278 (12)	0.0601 (8)	
C11	0.2522 (3)	0.1935 (2)	0.20299 (16)	0.0773 (10)	
C12	0.3485 (4)	0.2768 (2)	0.22841 (18)	0.0847 (12)	
C13	0.3378 (3)	0.3395 (2)	0.28381 (14)	0.0670 (8)	
C14	0.2302 (3)	0.3203 (2)	0.31371 (13)	0.0652 (8)	
C15	0.1327 (3)	0.23722 (19)	0.28775 (13)	0.0631 (8)	
C17	0.3273 (2)	−0.22232 (17)	0.45406 (12)	0.0563 (7)	
C18	0.3073 (3)	−0.20476 (18)	0.52750 (12)	0.0569 (7)	
C19	0.3666 (3)	−0.1188 (2)	0.57137 (15)	0.0762 (10)	
C20	0.3439 (4)	−0.1023 (3)	0.63736 (15)	0.0896 (11)	
C21	0.2629 (3)	−0.1724 (3)	0.66053 (16)	0.0884 (13)	
C22	0.2052 (3)	−0.2577 (3)	0.61814 (18)	0.0877 (11)	
C23	0.2268 (3)	−0.2744 (2)	0.55178 (16)	0.0725 (9)	
C24	0.4921 (2)	−0.25730 (17)	0.46936 (12)	0.0556 (7)	
C25	0.5204 (3)	−0.3596 (2)	0.46298 (15)	0.0710 (9)	
C26	0.6693 (4)	−0.3932 (2)	0.47724 (17)	0.0849 (11)	
C27	0.7892 (3)	−0.3270 (3)	0.49653 (15)	0.0819 (13)	
C28	0.7633 (3)	−0.2248 (3)	0.50267 (15)	0.0785 (10)	
C29	0.6157 (3)	−0.1903 (2)	0.48981 (14)	0.0700 (8)	
H2A	−0.02550	0.01470	0.33090	0.0770*	
H2B	0.14780	0.05190	0.37020	0.0770*	
H3A	0.11070	−0.08900	0.43590	0.0720*	
H3B	0.05410	−0.15440	0.36130	0.0720*	
H5A	0.22580	−0.20230	0.30600	0.0730*	
H5B	0.40200	−0.17110	0.34290	0.0730*	
H6A	0.33330	0.00080	0.31280	0.0760*	
H6B	0.27090	−0.06560	0.23850	0.0760*	
H11	0.26010	0.15100	0.16600	0.0930*	
H12	0.42120	0.29090	0.20820	0.1020*	
H14	0.22290	0.36260	0.35100	0.0780*	
H15	0.05880	0.22390	0.30740	0.0760*	
H17	0.25590	−0.27700	0.42630	0.0680*	
H19	0.42240	−0.07150	0.55640	0.0910*	
H20	0.38330	−0.04380	0.66600	0.1080*	
H21	0.24770	−0.16160	0.70490	0.1060*	
H22	0.15070	−0.30520	0.63380	0.1050*	
H23	0.18670	−0.33290	0.52340	0.0870*	
H25	0.43890	−0.40610	0.44900	0.0850*	
H26	0.68700	−0.46240	0.47350	0.1020*	
H27	0.88860	−0.35040	0.50560	0.0980*	
H28	0.84530	−0.17880	0.51550	0.0940*	
H29	0.59950	−0.12130	0.49500	0.0840*	

### Atomic displacement parameters (Å^2^)

**Table:**

	*U*^11^	*U*^22^	*U*^33^	*U*^12^	*U*^13^	*U*^23^
Cl16	0.0815 (5)	0.0828 (5)	0.1088 (6)	−0.0121 (4)	0.0313 (4)	0.0117 (4)
S7	0.0656 (4)	0.0731 (4)	0.0505 (4)	0.0021 (3)	0.0100 (3)	0.0025 (3)
O8	0.0547 (10)	0.0906 (13)	0.0809 (12)	0.0060 (9)	0.0079 (8)	0.0115 (10)
O9	0.1093 (14)	0.0931 (13)	0.0481 (9)	0.0001 (12)	0.0181 (9)	−0.0040 (10)
N1	0.0570 (11)	0.0656 (11)	0.0507 (10)	0.0035 (9)	0.0170 (8)	0.0024 (9)
N4	0.0527 (10)	0.0602 (11)	0.0488 (9)	0.0038 (8)	0.0199 (8)	0.0002 (8)
C2	0.0625 (13)	0.0731 (15)	0.0598 (13)	0.0111 (11)	0.0283 (11)	0.0045 (12)
C3	0.0561 (13)	0.0687 (14)	0.0586 (13)	0.0073 (11)	0.0258 (10)	0.0003 (12)
C5	0.0633 (14)	0.0705 (14)	0.0511 (12)	0.0099 (11)	0.0247 (10)	−0.0015 (11)
C6	0.0602 (14)	0.0752 (16)	0.0552 (13)	0.0064 (11)	0.0246 (10)	0.0004 (12)
C10	0.0624 (13)	0.0664 (14)	0.0488 (12)	0.0087 (11)	0.0196 (10)	0.0103 (11)
C11	0.0962 (19)	0.0804 (18)	0.0711 (16)	0.0040 (15)	0.0502 (15)	0.0042 (15)
C12	0.091 (2)	0.089 (2)	0.096 (2)	−0.0023 (16)	0.0603 (17)	0.0113 (18)
C13	0.0623 (14)	0.0679 (15)	0.0674 (15)	0.0039 (12)	0.0226 (12)	0.0163 (13)
C14	0.0719 (15)	0.0680 (15)	0.0563 (13)	0.0042 (12)	0.0264 (11)	0.0023 (12)
C15	0.0621 (14)	0.0718 (15)	0.0606 (13)	0.0049 (12)	0.0299 (11)	0.0057 (12)
C17	0.0547 (12)	0.0559 (12)	0.0539 (12)	−0.0037 (10)	0.0171 (9)	−0.0030 (10)
C18	0.0503 (12)	0.0650 (14)	0.0533 (12)	0.0016 (10)	0.0186 (9)	0.0083 (11)
C19	0.0865 (18)	0.0880 (18)	0.0602 (14)	−0.0231 (15)	0.0359 (13)	−0.0095 (14)
C20	0.093 (2)	0.118 (2)	0.0596 (15)	−0.0119 (18)	0.0325 (14)	−0.0179 (17)
C21	0.0703 (17)	0.139 (3)	0.0609 (15)	0.0153 (18)	0.0315 (13)	0.0205 (19)
C22	0.0742 (18)	0.112 (2)	0.090 (2)	0.0156 (17)	0.0467 (16)	0.040 (2)
C23	0.0617 (14)	0.0716 (16)	0.0852 (18)	0.0061 (12)	0.0305 (13)	0.0190 (14)
C24	0.0558 (12)	0.0585 (13)	0.0482 (11)	0.0026 (10)	0.0164 (9)	0.0009 (10)
C25	0.0751 (16)	0.0623 (14)	0.0759 (16)	0.0023 (12)	0.0306 (13)	−0.0057 (13)
C26	0.087 (2)	0.0782 (18)	0.090 (2)	0.0235 (16)	0.0360 (16)	−0.0004 (16)
C27	0.0639 (16)	0.113 (3)	0.0660 (16)	0.0199 (16)	0.0230 (13)	−0.0014 (16)
C28	0.0571 (15)	0.101 (2)	0.0690 (16)	−0.0054 (14)	0.0166 (12)	−0.0045 (15)
C29	0.0605 (14)	0.0712 (15)	0.0726 (15)	−0.0041 (12)	0.0207 (12)	−0.0055 (13)

### Geometric parameters (Å, °)

**Table:**

Cl16—C13	1.730 (3)	C25—C26	1.385 (5)
S7—O8	1.427 (2)	C26—C27	1.351 (5)
S7—O9	1.4261 (18)	C27—C28	1.376 (6)
S7—N1	1.637 (2)	C28—C29	1.383 (4)
S7—C10	1.761 (3)	C2—H2A	0.9692
N1—C2	1.473 (3)	C2—H2B	0.9704
N1—C6	1.470 (4)	C3—H3A	0.9701
N4—C3	1.466 (4)	C3—H3B	0.9700
N4—C5	1.468 (3)	C5—H5A	0.9704
N4—C17	1.479 (3)	C5—H5B	0.9705
C2—C3	1.508 (3)	C6—H6A	0.9699
C5—C6	1.511 (3)	C6—H6B	0.9687
C10—C11	1.376 (4)	C11—H11	0.9296
C10—C15	1.384 (3)	C12—H12	0.9296
C11—C12	1.377 (4)	C14—H14	0.9305
C12—C13	1.381 (4)	C15—H15	0.9302
C13—C14	1.372 (4)	C17—H17	0.9806
C14—C15	1.381 (4)	C19—H19	0.9291
C17—C18	1.515 (3)	C20—H20	0.9302
C17—C24	1.526 (3)	C21—H21	0.9305
C18—C19	1.384 (4)	C22—H22	0.9297
C18—C23	1.381 (4)	C23—H23	0.9291
C19—C20	1.385 (4)	C25—H25	0.9300
C20—C21	1.376 (5)	C26—H26	0.9306
C21—C22	1.362 (5)	C27—H27	0.9303
C22—C23	1.387 (4)	C28—H28	0.9303
C24—C25	1.383 (3)	C29—H29	0.9296
C24—C29	1.381 (4)		
			
Cl16···C26^i^	3.629 (3)	H2B···H6A	2.4989
Cl16···H6B^ii^	3.1028	H2B···H15	2.5367
Cl16···H20^iii^	2.9831	H3A···C18	2.4914
O8···H2A	2.4862	H3A···C19	2.7733
O8···H15	2.7200	H3B···H5A	2.3453
O8···H5A^iv^	2.8746	H3B···H17	2.4164
O8···H17^iv^	2.8583	H3B···C10^x^	3.0203
O8···H21^v^	2.6767	H3B···C15^x^	3.0457
O9···H6B	2.5606	H5A···H3B	2.3453
O9···H11	2.5312	H5A···H17	2.4216
N1···N4	2.865 (3)	H5A···O8^x^	2.8746
N4···N1	2.865 (3)	H5B···C24	2.5009
N4···H19	2.7600	H5B···C29	2.7426
N4···H29	2.7616	H5B···H12^ix^	2.2977
C2···C15	3.421 (4)	H6A···C10	2.9100
C3···C19	3.341 (4)	H6A···H2B	2.4989
C5···C29	3.326 (4)	H6A···C20^vii^	3.0905
C6···C11	3.588 (4)	H6A···H20^vii^	2.5882
C11···C6	3.588 (4)	H6B···O9	2.5606
C15···C2	3.421 (4)	H6B···Cl16^ix^	3.1028
C19···C29	3.428 (4)	H6B···H22^xi^	2.5247
C19···C3	3.341 (4)	H11···O9	2.5312
C26···Cl16^vi^	3.629 (3)	H11···C27^ii^	2.9834
C29···C19	3.428 (4)	H12···H5B^ii^	2.2977
C29···C5	3.326 (4)	H14···C26^vii^	3.0673
C2···H15	3.0460	H14···C27^vii^	3.0150
C10···H2B	3.0749	H15···O8	2.7200
C10···H6A	2.9100	H15···C2	3.0460
C10···H3B^iv^	3.0203	H15···H2B	2.5367
C15···H3B^iv^	3.0457	H17···H3B	2.4164
C15···H2B	2.8738	H17···H5A	2.4216
C18···H3A	2.4914	H17···H23	2.3276
C19···H29	3.0851	H17···H25	2.3258
C19···H3A	2.7733	H17···O8^x^	2.8583
C20···H6A^vii^	3.0905	H19···N4	2.7600
C21···H2A^v^	3.0936	H19···C29	3.0375
C23···H27^viii^	3.0998	H19···H29	2.4822
C24···H5B	2.5009	H20···H6A^vii^	2.5882
C26···H14^vii^	3.0673	H20···Cl16^xii^	2.9831
C27···H14^vii^	3.0150	H21···O8^v^	2.6767
C27···H11^ix^	2.9834	H22···H6B^xiii^	2.5247
C29···H5B	2.7426	H23···H17	2.3276
C29···H19	3.0375	H25···H17	2.3258
H2A···O8	2.4862	H27···C23^xiv^	3.0998
H2A···C21^v^	3.0936	H29···N4	2.7616
H2B···C10	3.0749	H29···C19	3.0851
H2B···C15	2.8738	H29···H19	2.4822
			
O8—S7—O9	119.92 (12)	C3—C2—H2B	109.79
O8—S7—N1	106.87 (11)	H2A—C2—H2B	108.30
O8—S7—C10	108.10 (13)	N4—C3—H3A	109.44
O9—S7—N1	107.06 (11)	N4—C3—H3B	109.43
O9—S7—C10	107.89 (12)	C2—C3—H3A	109.43
N1—S7—C10	106.23 (11)	C2—C3—H3B	109.45
S7—N1—C2	117.23 (16)	H3A—C3—H3B	108.00
S7—N1—C6	116.13 (16)	N4—C5—H5A	109.60
C2—N1—C6	110.86 (18)	N4—C5—H5B	109.59
C3—N4—C5	107.52 (18)	C6—C5—H5A	109.59
C3—N4—C17	110.91 (18)	C6—C5—H5B	109.52
C5—N4—C17	110.54 (17)	H5A—C5—H5B	108.06
N1—C2—C3	109.3 (2)	N1—C6—H6A	109.82
N4—C3—C2	111.0 (2)	N1—C6—H6B	109.82
N4—C5—C6	110.4 (2)	C5—C6—H6A	109.84
N1—C6—C5	109.2 (2)	C5—C6—H6B	109.84
S7—C10—C11	119.87 (19)	H6A—C6—H6B	108.34
S7—C10—C15	120.1 (2)	C10—C11—H11	120.23
C11—C10—C15	120.0 (2)	C12—C11—H11	120.31
C10—C11—C12	119.5 (3)	C11—C12—H12	119.84
C11—C12—C13	120.4 (3)	C13—C12—H12	119.79
Cl16—C13—C12	120.2 (2)	C13—C14—H14	120.52
Cl16—C13—C14	119.3 (2)	C15—C14—H14	120.37
C12—C13—C14	120.5 (3)	C10—C15—H15	119.73
C13—C14—C15	119.1 (2)	C14—C15—H15	119.73
C10—C15—C14	120.5 (3)	N4—C17—H17	107.75
N4—C17—C18	110.71 (18)	C18—C17—H17	107.76
N4—C17—C24	111.52 (17)	C24—C17—H17	107.73
C18—C17—C24	111.19 (18)	C18—C19—H19	119.61
C17—C18—C19	121.5 (2)	C20—C19—H19	119.51
C17—C18—C23	120.3 (2)	C19—C20—H20	120.01
C19—C18—C23	118.3 (2)	C21—C20—H20	119.98
C18—C19—C20	120.9 (3)	C20—C21—H21	120.20
C19—C20—C21	120.0 (3)	C22—C21—H21	120.20
C20—C21—C22	119.6 (3)	C21—C22—H22	119.68
C21—C22—C23	120.7 (3)	C23—C22—H22	119.65
C18—C23—C22	120.6 (3)	C18—C23—H23	119.71
C17—C24—C25	119.4 (2)	C22—C23—H23	119.74
C17—C24—C29	122.4 (2)	C24—C25—H25	119.78
C25—C24—C29	118.2 (2)	C26—C25—H25	119.83
C24—C25—C26	120.4 (2)	C25—C26—H26	119.51
C25—C26—C27	121.0 (3)	C27—C26—H26	119.53
C26—C27—C28	119.5 (3)	C26—C27—H27	120.19
C27—C28—C29	120.2 (3)	C28—C27—H27	120.26
C24—C29—C28	120.7 (3)	C27—C28—H28	119.90
N1—C2—H2A	109.75	C29—C28—H28	119.94
N1—C2—H2B	109.78	C24—C29—H29	119.65
C3—C2—H2A	109.89	C28—C29—H29	119.65
			
O8—S7—N1—C2	−42.1 (2)	C10—C11—C12—C13	0.5 (4)
O9—S7—N1—C2	−171.75 (18)	C11—C12—C13—C14	−0.7 (4)
C10—S7—N1—C2	73.2 (2)	C11—C12—C13—Cl16	179.6 (2)
O8—S7—N1—C6	−176.37 (16)	Cl16—C13—C14—C15	179.9 (2)
O9—S7—N1—C6	53.96 (19)	C12—C13—C14—C15	0.1 (4)
C10—S7—N1—C6	−61.13 (19)	C13—C14—C15—C10	0.5 (4)
O9—S7—C10—C11	−13.5 (2)	N4—C17—C18—C19	−48.4 (3)
N1—S7—C10—C11	101.0 (2)	C24—C17—C18—C23	−104.7 (3)
O8—S7—C10—C15	37.7 (2)	N4—C17—C24—C25	−135.1 (2)
O9—S7—C10—C15	168.8 (2)	N4—C17—C24—C29	45.2 (3)
N1—S7—C10—C15	−76.7 (2)	C18—C17—C24—C25	100.8 (2)
O8—S7—C10—C11	−144.6 (2)	C18—C17—C24—C29	−78.9 (3)
S7—N1—C2—C3	166.58 (18)	N4—C17—C18—C23	130.7 (2)
C6—N1—C2—C3	−56.9 (3)	C24—C17—C18—C19	76.1 (3)
C2—N1—C6—C5	57.6 (2)	C19—C18—C23—C22	0.6 (4)
S7—N1—C6—C5	−165.36 (15)	C17—C18—C23—C22	−178.6 (3)
C3—N4—C5—C6	61.4 (3)	C17—C18—C19—C20	178.2 (3)
C17—N4—C3—C2	178.14 (18)	C23—C18—C19—C20	−0.9 (4)
C5—N4—C3—C2	−60.9 (2)	C18—C19—C20—C21	0.7 (5)
C3—N4—C17—C24	177.36 (17)	C19—C20—C21—C22	−0.1 (5)
C17—N4—C5—C6	−177.4 (2)	C20—C21—C22—C23	−0.2 (5)
C3—N4—C17—C18	−58.3 (2)	C21—C22—C23—C18	0.0 (5)
C5—N4—C17—C18	−177.4 (2)	C17—C24—C25—C26	−179.4 (2)
C5—N4—C17—C24	58.2 (2)	C25—C24—C29—C28	0.7 (4)
N1—C2—C3—N4	59.0 (3)	C29—C24—C25—C26	0.3 (4)
N4—C5—C6—N1	−60.3 (3)	C17—C24—C29—C28	−179.5 (2)
S7—C10—C11—C12	−177.6 (2)	C24—C25—C26—C27	−1.0 (4)
C15—C10—C11—C12	0.1 (4)	C25—C26—C27—C28	0.5 (4)
S7—C10—C15—C14	177.0 (2)	C26—C27—C28—C29	0.6 (4)
C11—C10—C15—C14	−0.6 (4)	C27—C28—C29—C24	−1.2 (4)

Symmetry codes: (i) *x*, *y*+1, *z*; (ii) −*x*+1, *y*+1/2, −*z*+1/2; (iii) *x*, −*y*+1/2, *z*−1/2; (iv) −*x*, *y*+1/2, −*z*+1/2; (v) −*x*, −*y*, −*z*+1; (vi) *x*, *y*−1, *z*; (vii) −*x*+1, −*y*, −*z*+1; (viii) *x*−1, *y*, *z*; (ix) −*x*+1, *y*−1/2, −*z*+1/2; (x) −*x*, *y*−1/2, −*z*+1/2; (xi) *x*, −*y*−1/2, *z*−1/2; (xii) *x*, −*y*+1/2, *z*+1/2; (xiii) *x*, −*y*−1/2, *z*+1/2; (xiv) *x*+1, *y*, *z*.

### Hydrogen-bond geometry (Å, °)

**Table:**

*D*—H···*A*	*D*—H	H···*A*	*D*···*A*	*D*—H···*A*
C2—H2A···O8	0.97	2.49	2.890 (3)	105
C6—H6B···O9	0.97	2.56	2.965 (3)	105
C11—H11···O9	0.93	2.53	2.905 (3)	104

## References

[bb1] Bassindale, A. (1984). *The Third Dimension in Organic Chemistry*, ch. 1, p. 11. New York: John Wiley & Sons.

[bb2] Berkheij, M., van der Sluis, L., Sewing, C., den Boer, D. J., Terpstra, J. W., Heimstra, H., Bakker, W. I. I., van den Hoogen Band, A. & van Maarseveen, J. H. (2005). *Tetrahedron*, **46**, 2369–2371.

[bb4] Campbell, H., Cline, W., Evans, M., Lloyd, J. & Peck, A. W. (1973). *Eur. J. Clin. Pharmacol.* **6**, 170–176. 10.1007/BF00558281 4586849

[bb5] Cremer, D. & Pople, J. A. (1975). *J. Am. Chem. Soc.* **97**, 1354–1358.

[bb6] Dinsmore, C. J. & Beshore, D. C. (2002). *Tetrahedron*, **58**, 3297–3312.

[bb7] Humle, C. & Cherrier, M. P. (1999). *Tetrahedron Lett.* **40**, 5295–5299.

[bb8] Johnson, C. K. (1976). *ORTEPII* Report ORNL-5138. Oak Ridge National Laboratory, Tennessee, USA.

[bb9] Katzung, B. G. (1995). *Basic and Clinical Pharmacology*, 6th ed. San Francisco: University of California.

[bb10] MacScience (2002). *XPRESS* MacScience Co. Ltd, Yokohama, Japan.

[bb11] Otwinowski, Z. & Minor, W. (1997). *Methods in Enzymology*, Vol. 276, *Macromolecular Crystallography*, Part A, edited by C. W. Carter Jr & R. M. Sweet, pp. 307–326. New York: Academic Press.

[bb12] Sheldrick, G. M. (1997). *SHELXS97* and *SHELXL97* University of Göttingen, Germany.

[bb13] Spek, A. L. (2003). *J. Appl. Cryst.* **36**, 7–13.

